# Computational phenotyping of obstructive airway diseases: protocol for a systematic review

**DOI:** 10.1186/s13643-022-02078-0

**Published:** 2022-10-13

**Authors:** Muwada Bashir Awad Bashir, Rani Basna, Guo-Qiang Zhang, Helena Backman, Anne Lindberg, Linda Ekerljung, Malin Axelsson, Linnea Hedman, Lowie Vanfleteren, Bo Lundbäck, Eva Rönmark, Bright I. Nwaru

**Affiliations:** 1 Krefting Research Centre, Institute of Medicine, University of Gothenburg, SE-405 30 Gothenburg, Sweden; 2 Section of Sustainable Health/the OLIN Unit, Department of Public Health and Clinical Medicine, Umeå University, Umeå, Sweden; 3 Section of Medicine/the OLIN Unit, Department of Public Health and Clinical Medicine, Umeå University, Umeå, Sweden; 4 Department of Care Science, Faculty of Health and Society, Malmö University, Malmö, Sweden; 5 Department of Health Sciences, Luleå University of Technology, Luleå, Sweden; 6 COPD Center, Sahlgrenska University Hospital, University of Gothenburg, Gothenburg, Sweden; 7 Wallenberg Centre for Molecular and Translational Medicine, Institute of Medicine, University of Gothenburg, Gothenburg, Sweden

**Keywords:** Airway disease, Asthma, Clustering, COPD, Computation, Machine learning, Phenotype, Systematic review

## Abstract

**Background:**

Over the last decade, computational sciences have contributed immensely to characterization of phenotypes of airway diseases, but it is difficult to compare derived phenotypes across studies, perhaps as a result of the different decisions that fed into these phenotyping exercises. We aim to perform a systematic review of studies using computational approaches to phenotype obstructive airway diseases in children and adults.

**Methods and analysis:**

We will search PubMed, Embase, Scopus, Web of Science, and Google Scholar for papers published between 2010 and 2020. Conferences proceedings, reference list of included papers, and experts will form additional sources of literature. We will include observational epidemiological studies that used a computational approach to derive phenotypes of chronic airway diseases, whether in a general population or in a clinical setting. Two reviewers will independently screen the retrieved studies for eligibility, extract relevant data, and perform quality appraisal of included studies. A third reviewer will arbitrate any disagreements in these processes. Quality appraisal of the studies will be undertaken using the Effective Public Health Practice Project quality assessment tool. We will use summary tables to describe the included studies. We will narratively synthesize the generated evidence, providing critical assessment of the populations, variables, and computational approaches used in deriving the phenotypes across studies

**Conclusion:**

As progress continues to be made in the area of computational phenotyping of chronic obstructive airway diseases, this systematic review, the first on this topic, will provide the state of the art on the field and highlight important perspectives for future works.

**Ethics and dissemination:**

No ethical approval is needed for this work is based only on the published literature and does not involve collection of any primary or human data.

**Registration and reporting:**

**Systematic review registration:**

PROSPERO CRD42020164898

**Supplementary Information:**

The online version contains supplementary material available at 10.1186/s13643-022-02078-0.

## Background

Asthma and chronic obstructive pulmonary diseases (COPD) are the most common chronic respiratory diseases worldwide, largely accounting for global mortality and morbidity burden [1, 2]. While one-fifth of the developed world population is expected to have asthma at certain time in their life especially in Europe [3], globally around 10% of adults currently have COPD [4]. By 2030, COPD is projected to be the fourth leading cause of death globally [5]. Other airway diseases, such as sinusitis and allergic rhinitis, although of lesser contribution to overall mortality, collectively can affect around 10–30% of the populations of western countries [4, 6]. They also account for significant loss in societal productivity due to loss of working and schooling hours and treatment expenditure [7, 8].

Over the last decade, significant progress has been made regarding improving understanding of the pathophysiological and clinical features of obstructive airway diseases. Indeed, we know today that diseases such as asthma and COPD are not single disease entities as previously thought; rather, they are heterogeneous in nature and embedded with varied underlying phenotypes [9, 10]. A phenotype is “the observable and structural and functional characteristics of an organism determined by its genotype and modulated by its environment” [11]. Better understanding of the phenotypes of airway diseases will provide the opportunity for targeted, individualized, and precise management of these diseases [12].

Generally, disease phenotyping falls into two areas: hypothesis-led approach and data-driven or computational approach. The hypothesis-led phenotyping relies on classifying diseases on the basis of the characteristics of the presenting patient, and the general framework has been to rely on the clinical or physiological features, based on specific triggers and pathobiology of inflammation [11, 13]. As no standard exists in such classifications, the clinician relies on the current knowledge of the disease and his own experiences and presumptions; consequently, the hypothesis-led approach is said to be largely subjective and may be potentially biased [14, 15]. The data-driven approach to phenotyping works through development of high-level computer algorithms that automatically learn from data and try to uncover complex patterns in a systematic and meaningful way [16]. Usually, no a priori theory is employed in learning from the data; rather, the computer allows the data to “speak for itself” and uncover hidden nuances that will enhance understanding and clinical decisions; consequently, the data-driven approach to phenotyping is said to be unbiased [16]. The advancement in machine-led computations and novel statistical methods in human diseases has facilitated the progress now being made in data-driven phenotyping of chronic obstructive airway diseases [17]. While the traditional clustering technique, like hierarchical clustering and partitioning methods, has remained the most frequently used conventional approach to disease phenotyping, several emerging machine-learning approaches, such as deep learning and probabilistic modelling, are providing advanced flavor to the phenotyping exercises [13].

Despite the progress now being made through use of these suits of computational approaches to uncover salient underlying phenotypes of obstructive airway diseases, a unified understanding of the available approaches remains uncertain. Each method appears to have unique underlying mathematical approach, which consequently influences their operations on the data fed into them and the eventual phenotypes derived. The rapid developments and variations in the computational approaches have meant that choosing from available approaches can be challenging. While several computational phenotyping studies of chronic obstructive airway diseases have been undertaken during the past decade [18–21], both in children and adults, replication of derived phenotypes across contexts and thus evaluating the clinical relevance of emanating phenotypes are unclear. There is therefore the need to undertake a systematic synthesis of the body of work so far undertaken in this area. Such an exercise will give researchers greater appreciation of the current state of the art, help to interpret the results that have emanated and evaluate their clinical relevance, and guide future works in this area [18, 20]. Furthermore, a systematic survey of the field of computational phenotyping of chronic airway diseases will help uncover the various choices that have been implemented in these exercises, including the characteristics of the population phenotyped, relevant inclusion criteria used, and variables included for deriving the phenotypes.

Given the uncertainty of the underlying evidence and the rapid progress being made, the aim of this study is to identify, critically appraise, and synthesize data from studies that have so far used computational approaches to phenotype chronic obstructive airway diseases in children and adults. Specifically, we aim the following:

1. Characterize and compare the populations included in studies of computational phenotyping of chronic airway diseases.
2. Assess and compare the criteria used to select participants included in studies of computational phenotyping of chronic airway diseases.
3. Evaluate and compare the variables used to derive phenotypes of chronic airway diseases across studies and assess the choices informing the included variables.
4. Describe and compare the computational approaches used across studies and highlight the features of each computational approach.
5. Describe the number and characteristics of phenotypes derived across studies and assess their clinical interpretation.

## Methods

### Eligibility criteria

We will include population-based studies that have used computational approaches to derive phenotypes of chronic airway diseases, whether conducted in the general population or in a clinical setting. We will exclude studies that have characterized phenotypes of chronic airway diseases based on hypothesis-based approaches.

### Study design

We will include observational general population-based and clinical epidemiological studies, including cohort, case control, and cross sectional. We do not anticipate computational phenotyping studies of airway diseases based on randomized clinical trials or other experimental study designs. Case studies and case series as well as ecological studies will be excluded.

### Participants

We will include studies conducted both in children and adults.

### Years of consideration

Studies conducted in the last 10 years (2010–2020) only will be considered for our review. The selected time window is the reported era of evolution of the use of computational approaches in phenotyping of chronic obstructive airway diseases [22].

### Language

There will be no language-based exclusions of studies, and we will endeavor to translate studies published in languages other than English.

### Information source

To identify relevant studies for the review, we will search PubMed, Embase, Web of Science, Scopus, and Google Scholar. For unpublished materials, such as conference proceedings, we will search databases of proceeding of conferences and databases of the gray literature, such as Open Grey. We will also contact experts in the field to request for any paper we may miss from our database searches. Finally, we will screen the reference lists of included studies to identify any additional paper.

### Search strategy

We have developed a preliminary search strategy to identify relevant studies for the review. The search strategy (Supplementary file 1) was developed in PubMed and will be adapted in searching the other databases.

### Study records

#### Data management and selection process

The search results from the different databases will be exported to EndNote for further screening. Two reviewers will independently screen the studies on the basis of the review inclusion and exclusion criteria; any discrepancies will be resolved by discussion, or a third reviewer will arbitrate if a consensus is not reached. The first stage of the literature will involve removal of duplicates from the database searches; then, we will perform title and abstract screening. The final stage will involve full-text screening of the studies potentially meeting the eligibility criteria not clearly identified from the titles and abstracts. We will document the screening process using the Preferred Reporting Items for Systematic Reviews and Meta-Analyses (PRISMA) flowchart [23].

#### Data collection process

Two reviewers will independently extract relevant data from included studies onto a data extraction form to be developed for the review; any discrepancies will be resolved by discussion, or a third reviewer will arbitrate if a consensus is not reached. We will develop a data extraction form specifically designed for this review that will be used to capture relevant data from included studies. The form will initially be first piloted on two to three included studies; any amendment will be undertaken prior to using the form on all included studies.

#### Data items

Information on the following data items will be collected from included studies into the data extraction form: general information (author’s name, publication year and study time, aim of the study, and data source); information describing populations characteristics (population size, recruitment characteristics, sample size, children/adults, inclusion and exclusions criteria); type of airway disease; information about the variables selected for phenotyping (number and description of variables, rational of selection, variable measurement and definition); type and features of computational approach used; and information of the derived phenotypes (number of phenotypes, characteristics of each phenotype, and clinical interpretation).

### Outcome and prioritization

We will include studies focusing on computational phenotyping of the following chronic obstructive airway diseases:

- Asthma
- COPD
- Rhinitis
- Emphysema

### Quality assessment of included studies

We will appraise the general quality of included studies using the Effective Public Health Practice Project (EPHPP), where the focus of this tool will be sorting studies in relation to each study’s potential for selection bias, appropriateness of study design, data collection methods, withdrawals and dropouts, and analysis [24]. Since, to our knowledge, there are no standard tools for assessing the quality of studies on computational disease phenotyping, we will develop a preliminary checklist that will enable us to extract items related to the computational approaches used across studies and to help us compare approaches across studies.

### Data synthesis

We will tabulate all data items extracted from studies, where a detailed descriptive narrative summary for each included study will be synthesized and presented. We do not aim to perform any quantitative summary (meta-analysis) for included studies as this is not the goal of the current work. However, we will employ a narrative synthesis of the underlying evidence, focusing at least on the following aspects: strengths, limitations of the included studies and features of the computational approaches used, description and comparison of the derived phenotypes across studies and their clinical relevance, description and comparison of the variables used for phenotyping and the populations characteristics in each study set up, and choices informing their consideration; issues of reproducibility of each phenotyping exercises; etc. [25].

## Discussion

The findings derived until date from studies using computational methods to phenotype chronic airway diseases have highlighted the importance of using these methods in delineating the heterogeneous nature of these diseases [14, 21, 26–28]. Still, the question about the reproducibility and clinical relevance of derived phenotypes remains a valid one. Factors of population characteristics, variables used to derive disease phenotypes, computational approaches used, and characteristics of derived phenotypes and their comparability across studies are issues that demand further scrutiny.

The current review, the first on the topic, to our knowledge, is an attempt to address these overarching issues. Findings from the review will therefore contribute in advancing the field of computational phenotyping of chronic obstructive airway diseases.

## Conclusion

As progress continues to be made in the area of computational phenotyping of chronic obstructive airway diseases, systematically surveying the field and appraising the evidence so far generated will help identify potential research gaps and how to fill them. The evidence to be generated from the current systematic review will therefore provide the current state of the art on the field and will highlight important perspectives for future works. This synthesis will give researchers in the area an accessible summary to guide their works in the use of computational approaches to phenotype chronic airway diseases.

## Supplementary Information

**Additional file 1:** **Supplementary file 1.** Supplemental: Databases search strategies.

## Data Availability

The data and articles used in this review, along with the analysis codes, will be availed through repository sets that will be generated during the current study.

## Abbreviations

COPD: Chronic obstructive pulmonary disease
PRISMA: Preferred Reporting Items for Systematic Reviews and Meta-Analyses
EPHPP: Effective Public Health Practice Project
