# Supplementary material for: Computational phenotyping of obstructive airway diseases: protocol for a systematic review
Source: Syst Rev. 2022 Oct 13;11:216. doi: 10.1186/s13643-022-02078-0 (PMC9559879; doi:10.1186/s13643-022-02078-0)
Supplement: Supplementary file 1 — Additional file 1: Supplementary file 1. Supplemental: Databases search strategies. [file 13643_2022_2078_MOESM1_ESM.pdf]

Computational phenotyping of obstructive airway diseases: protocol for a systematic review

Supplemental: Databases search strategies

	**PubMed 2020-01-14**	
#1	natural language processing[mesh] OR natural language processing[tiab] OR Hierarchical clustering[tiab] OR cluster analysis[mesh] OR cluster analysis[tiab] OR cluster analyses[tiab] OR deep learning[mesh] OR deep learning[tiab] OR hierarchial learning[tiab] OR machine learning[mesh] OR machine learning[tiab] OR artificial intelligence[mesh] OR artificial intelligence[tiab] OR AI[tiab] OR computational intelligence[tiab] OR Self-organizing map*[tiab] OR Kohonen map*[tiab] OR Latent Class Analysis[Mesh] OR latent class analysis[tiab] OR latent class[tiab] OR latent growth model[tiab] OR latent growth modelling[tiab] OR latent growth models[tiab] OR latent class model[tiab] OR latent class models[tiab] OR latent class modelling[tiab] OR latent transition analysis[tiab] OR latent transition model[tiab] OR latent transition models[tiab] OR latent transition modelling[tiab] OR latent growth analysis[tiab] OR latent growth curve[tiab] OR latent growth curves[tiab] OR latent growth curve analysis[tiab] OR latent growth curve model[tiab] OR latent growth curve models[tiab] OR latent growth curve modelling[tiab] OR k-means[tiab] OR k-means clustering[tiab] OR k-means clusters[tiab]	221 182
#2	Phenotyp*[tiab] OR phenotype[mesh]	699 338
#3	Computational[tiab] OR automat*[tiab] OR unsupervised[tiab]	361 595
#4	#1 OR (#2 AND #3)	230 094
#5	Lung Diseases, Obstructive[mesh] OR emphysema*[tiab] OR asthma[tiab] OR rhinitis[mesh] OR rhinitis[tiab] OR chronic obstructive pulmonary disease[tiab] OR COPD[tiab] OR COAD[tiab] OR COBD[tiab] OR respiratory sounds[MeSH Terms] OR respiratory sound*[tiab] OR wheeze[tiab] OR wheezing[tiab] OR wheezy[tiab] OR asthma[MESH Terms] OR pulmonary disease, chronic obstructive[MeSH Terms] OR rhinosinusitis[tiab]	323 000
#6	#4 AND #5	1 983
#7	Child[ti] OR children[ti] OR pediatric*[ti] OR paediatric*[ti] OR infant*[ti]	924 253
#8	(child[mesh]) NOT (child[mesh] AND adult[mesh])	1 187 301
#9	#7 OR #8	1 567 617
#10	#6 NOT #9	1 652

	**Scopus 2020-01-14**	
#1	TITLE-ABS-KEY (“natural language processing” OR “Hierarchical clustering” OR “cluster analysis” OR “cluster analyses” OR “deep learning” OR “hierarchial learning” OR “machine learning” OR “artificial intelligence” OR AI OR “computational intelligence” OR "self-organizing map*” OR “Kohonen map*” OR “latent class” OR “latent growth model*” OR “latent transition analysis” OR “latent transition analyses” OR “latent transition model*” OR “latent growth analysis” OR “latent growth analyses” OR “latent growth curve*” OR k-means)	829639
#2	TITLE-ABS-KEY(phenotyp*)	805611
#3	TITLE-ABS-KEY(computational OR automat* OR unsupervised)	2582042
#4	#2 AND #3	17964
#5	#4 OR #1	845314
#6	TITLE-ABS-KEY(emphysema* OR asthma OR rhinitis OR "chronic obstructive pulmonary disease” OR copd OR coad OR cobd OR "respiratory sound*" OR wheeze OR wheezing OR wheezy OR rhinosinusitis)	410821
#7	#6 AND #5	2590
#8	TITLE(child OR children OR pediatric* OR paediatric* OR infant*)	
#9	#7 AND NOT #8	2383
#10	#9 AND NOT index(medline)	655

	**WoS 2020-02-14**	
#1	TOPIC: (“natural language processing” OR “Hierarchical clustering” OR “cluster analysis” OR “cluster analyses” OR “deep learning” OR “hierarchial learning” OR “machine learning” OR “artificial intelligence” OR AI OR “computational intelligence” OR "self-organizing map*” OR “Kohonen map*” OR “latent class” OR “latent growth model*” OR “latent transition analysis” OR “latent transition analyses” OR “latent transition model*” OR “latent growth analysis” OR “latent growth analyses” OR “latent growth curve*” OR k-means)	347105
#2	TOPIC: (phenotyp*)	629351
#3	TOPIC: (computational OR automat* OR unsupervised)	1447833
#4	#2 AND #3	10348
#5	#4 OR #1	356395
#6	TOPIC: (emphysema* OR asthma OR rhinitis OR "chronic obstructive pulmonary disease” OR copd OR coad OR cobd OR "respiratory sound*" OR wheeze OR wheezing OR wheezy OR rhinosinusitis)	290951
#7	#6 AND #5	1847
#8	TITLE: (child OR children OR pediatric* OR paediatric* OR infant*)	
#9	#7 NOT #8	1698

Number	Google scholar 2020-1-21	
**#1**	(“natural language processing”OR“Hierarchical clustering”OR“cluster analysis”OR“cluster analyses”OR“deep learning”OR“hierarchical learning”OR“machine learning”OR“artificial intelligence” OR AI OR “computational intelligence” OR "self-ORganizing map” OR “Kohonen map” OR “latent class” OR “latent growth model” OR “latent transition analysis” OR “latent transition analyses” OR “latent transition model” OR “latent growth analysis” OR “latent growth analyses” OR “latent growth curve” OR k-means)	17100
**#2**	Allintitle: (“natural language processing” OR “Hierarchical clustering” OR “cluster analysis” OR “cluster analyses” OR “deep learning” OR “hierarchial learning” OR “machine learning” OR “artificial intelligence” OR AI OR “computational intelligence” OR "self-organizing map” OR “Kohonen map” OR “latent class” OR “latent growth model” OR “latent transition analysis” OR “latent transition analyses” OR “latent transition model” OR “latent growth analysis” OR “latent growth analyses” OR “latent growth curve” OR k-means)	184
**#3**	(phenotype)	1290000
**#4**	Allintitle: (phenotype)	15
**#5**	(computational OR automated OR unsupervised)	1360000
**#6**	Allintitle: (computational OR automated OR unsupervised)	196
**#7**	**#3 AND #5**	352000
**#3 and #5**	(computational OR automated OR unsupervised) AND (phenotype)	352000
**#8**	(emphysema OR asthma OR rhinitis OR "chronic obstructive pulmonary disease” OR copd OR coad OR cobd OR "respiratory sound*" OR wheeze OR wheezing OR wheezy OR rhinosinusitis)	238 000
**#9**	**#1 AND (#3AND #5 AND #7)**	982
**#1 AND (#3AND #5 AND #7)**	((“natural language processing”OR“Hierarchical clustering”OR“cluster analysis”OR“cluster analyses”OR“deep learning”OR“hierarchical learning”OR“machine learning”OR“artificial intelligence” OR AI OR “computational intelligence” OR"self-organizing map” OR “Kohonen map” OR “latent class” OR “latent growth model” OR “latent transition analysis” OR “latent transition analyses” OR “latent transition model” OR “latent growth analysis” OR “latent growth	982

	analyses" OR "latent growth curve" OR k-means) AND ((phenotype) AND (computational OR automated OR unsupervised)) AND (emphysema OR asthma OR rhinitis OR "chronic obstructive pulmonary disease" OR COPD OR COPD OR COPD OR "respiratory sound*" OR wheeze OR wheezing OR wheezy OR rhinosinusitis))	

EMBASE : 20th January 2020

1. natural language processing.mp. or *natural language processing/
2. exp cluster analysis/ or Hierarchical clustering.mp.
3. deep learning.mp. or exp deep learning/
4. machine learning.mp. or exp machine learning/
5. artificial intelligence.mp. or exp artificial intelligence/
6. *mathematical computing/ or computational intelligence.mp.
7. Self-organizing map*.mp.
8. Kohonen map*.mp.
9. Latent Class Analysis.mp. or exp latent class analysis/
10. latent class*.mp.
11. latent growth model*.mp.
12. latent class model*.mp.
13. latent transition analysis.mp.
14. latent transition model*.mp.
15. latent growth analysis.mp.
16. latent growth curve.mp.
17. latent growth curves.mp.
18. latent growth curve analysis.mp.
19. latent growth curve model*.mp.
20. k-means.mp.
21. k-means cluster*.mp.
22. 1 or 2 or 3 or 4 or 5 or 6 or 7 or 8 or 9 or 10 or 11 or 12 or 13 or 14 or 15 or 16 or 17 or 18 or 19 or 20 or 21
23. exp phenotype/ or Phenotyp*.mp.
24. computational.mp.
25. automat*.mp. or exp automation/
26. unsupervised.mp. or exp unsupervised machine learning/
27. 24 or 25 or 26
28. 23 and 27
29. 22 or 28
30. obstructive lung disease.mp. or exp chronic obstructive lung disease/
31. obstructive airway disease.mp. or exp obstructive airway disease/

32. emphysema*.mp. or exp lung emphysema/ or exp emphysema/
33. exp asthma/ or asthma.mp.
34. chronic obstructive pulmonary disease.mp. or exp chronic obstructive lung disease/
35. copd.mp.
36. coad.mp.
37. wheeze.mp. or exp wheezing/
38. exp wheezing/ or wheeze*.mp.
39. exp chronic rhinosinusitis/ or rhinosinusitis.mp. or *rhinosinusitis/
40. 30 or 31 or 32 or 33 or 34 or 35 or 36 or 37 or 38 or 39
41. 29 and 40
